# Linking Epigenetics to Human Disease and Rett Syndrome: The Emerging Novel and Challenging Concepts in MeCP2 Research

**DOI:** 10.1155/2012/415825

**Published:** 2012-02-09

**Authors:** Robby Mathew Zachariah, Mojgan Rastegar

**Affiliations:** ^1^Regenerative Medicine Program, University of Manitoba, 745 Bannatyne Avenue, Winnipeg, MB, Canada R3E 0J9; ^2^Department of Biochemistry and Medical Genetics, University of Manitoba, 745 Bannatyne Avenue, Winnipeg, MB, Canada R3E 0J9; ^3^Department of Immunology, University of Manitoba, 745 Bannatyne Avenue, Winnipeg, MB, Canada R3E 0J9

## Abstract

Epigenetics refer to inheritable changes beyond DNA sequence that control cell identity and morphology. Epigenetics play key roles in development and cell fate commitments and highly impact the etiology of many human diseases. A well-known link between epigenetics and human disease is the X-linked *MECP2* gene, mutations in which lead to the neurological disorder, Rett Syndrome. Despite the fact that MeCP2 was discovered about 20 years ago, our current knowledge about its molecular function is not comprehensive. While MeCP2 was originally found to bind methylated DNA and interact with repressor complexes to inhibit and silence its genomic targets, recent studies have challenged this idea. Indeed, depending on its interacting protein partners and target genes, MeCP2 can act either as an activator or as a repressor. Furthermore, it is becoming evident that although Rett Syndrome is a progressive and postnatal neurological disorder, the consequences of MeCP2 deficiencies initiate much earlier and before birth. To comprehend the novel and challenging concepts in MeCP2 research and to design effective therapeutic strategies for Rett Syndrome, a targeted collaborative effort from scientists in multiple research areas to clinicians is required.

## 1. Introduction

The term epigenetics refers to inheritable changes in gene expression that control cellular phenotype and fate decisions without alterations in the underlying DNA sequence [1]. In eukaryotes, two main epigenetic regulations are exerted through modifications on DNA and DNA-bound histone proteins. In general, histone modifications are dynamic and include acetylation, methylation, isomerization, phosphorylation, sumoylation, and ubiquitination [1, 2]. The combination of such modifications confers enormous flexibility in terms of functional response of an individual cell towards extracellular signals and environmental stimuli. Certain modifications such as histone methylation can display additional layers of complexity regarding their methylation degree and undergo mono-, di-, or tri-methylation of lysine residues [2, 3]. Furthermore, combinations or sequential additions of different histone marks can affect the chromatin organization and subsequently alter the expression of the corresponding target genes [4]. Conventionally, DNA methylation was considered to be a stable epigenetic mark, although this notion is being challenged by recent reports of active DNA demethylation [5]. In mammals, DNA methylation strictly happens at the cytosine residues in the context of CpG dinucleotides. The methylation of DNA molecules is processed by a group of enzymes called DNA methyltransferases (DNMTs). The mammalian DNMT family consists of 5 proteins (DNMT1, 2, 3A, 3B, 3L). DNMT1 is involved in maintaining the DNA methylation pattern during replication, while DNMT3A and DNMT3B act as *de novo* methyltransferases. DNMT3L is essential for the establishment of maternal genomic imprints during oocyte development, and DNMT2 is classified as part of the DNMT family; however it has very weak catalytic activity [6]. 

DNA methylation is often associated with transcriptional repression and has been linked to the tissue-specific regulation of genes [7], expression of imprinted genes [8], and X-chromosome inactivation in females [9]. In general, DNA methylation affects gene expression in two ways: (i) directly, by altering the binding sites of transcription factors, or (ii) indirectly, *via *recruitment of proteins that recognize and bind to the methylated DNA and in turn modulate gene expression. The first group of proteins that were discovered with the potential of binding to methylated DNA were the MBD (methyl-binding domain) protein family members. The mammalian MBD family consists of 5 nuclear proteins, MBD 1–4 and MeCP2 (Methyl CpG binding protein 2). With the exception of MBD3, all MBD proteins share a conserved methyl-binding domain, through which they bind to methylated DNA [10]. MBD3 lacks such ability due to a critical mutation in its MBD domain [11]. *MECP*2 is an X-linked gene, which was discovered as the prototype member of the DNA methyl binding proteins (MBPs) [12]. Mutations in *MECP2 *are the primary cause of Rett Syndrome (RTT), a neurological disorder predominantly affecting young females. RTT is characterized by an apparently normal development for the first 6–18 months after birth, followed by regression of acquired motor and language skills [13–15]. In addition to Rett Syndrome, mutations in *MECP2* have been observed in patients with classical autism, neonatal encephalopathy, and X-linked mental retardation [16–19]. 

**Figure 1:**
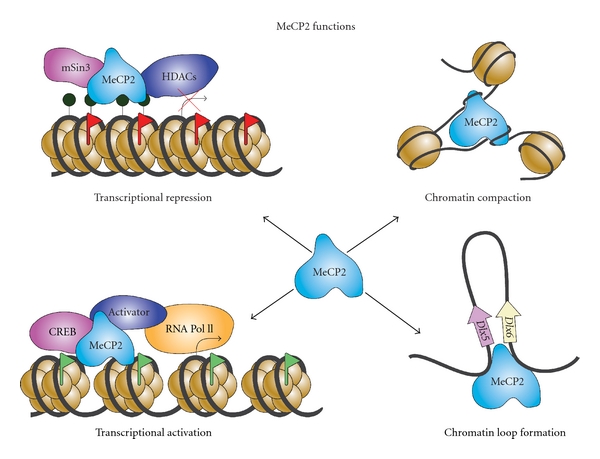
The diverse functions of MeCP2 in gene regulation and chromatin organization.

Studies on MeCP2 have yielded surprising results in terms of the diversity of its functions (Figure 1) with enormous potential for epigenetic regulation of target gene expression. MeCP2 was initially identified as a methyl-binding protein [20]. Further investigations on MeCP2 function led to the discovery of its role as a transcriptional repressor and association with corepressor complexes such as mSin3A and HDACs [21, 22]. This was not surprising, since DNA methylation itself was considered to be a repressive mark. However, a genomewide search for MeCP2 genomic distribution in SH-SY5Y cells led to two surprising observations: (i) MeCP2 was found to be associated often with transcriptionally active genes; (ii) only 2.2% of the most methylated promoters were bound by MeCP2. The presence of MeCP2 at the active promoters was later observed in mouse hypothalamus, where MeCP2 was observed to be bound to approximately 85% of genes which were misregulated by overexpression or absence of MeCP2 [23]. These studies highlight the many facets of MeCP2 functions and emphasize the need to further study its known functions. In this review, we will discuss the role of MeCP2 in chromatin structure and nuclear architecture of neurons, its competition with the linker histone H1, the *MECP2* transcript products and diverse functional domains of MeCP2 protein, as well as MeCP2 expression and genomic targets in neurons and glia. 

## 2. The *MECP2* Gene Structure and Its Splice Variants

**Figure 2:**
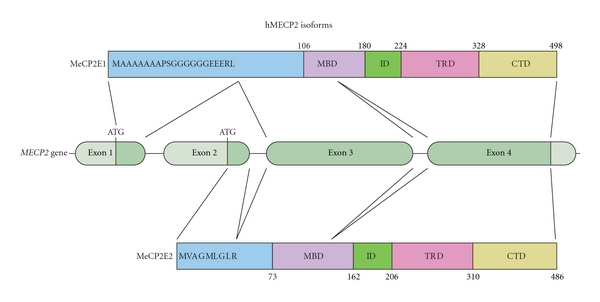
*MECP2* gene and protein isoforms. Schematic illustration of the gene structure of *MECP2* and the different domains of the two protein isoforms, MeCP2E1 and MeCP2E2. The primary amino acid composition of the N-terminus of MeCP2E1 and MeCP2E2 is depicted.

The *MECP2* gene maps between *L1CAM* and the *RCP/GCP* loci in Xq28 and undergoes X-Chromosome Inactivation (XCI) in females [24, 25]. The genomic locus of *MECP2* spans approximately 76 kb and consists of four exons encoding two different isoforms (MeCP2E1 and MeCP2E2), due to alternate splicing of exon 2 (Figure 2). The more abundant E1 isoform contains 24 amino acids encoded by exon 1 and lacks the 9 amino acids encoded by exon 2, whereas the start site for the E2 lies within the exon 2 [26]. Of the two isoforms, *MECP2E1* is more efficiently translated and show 10X more expression than *MECP2E2* in brain. *Mecp2* has a large, highly conserved 3′UTR that contains multiple polyadenylation sites. Alternative 3′UTR usage leads to three distinct transcripts, short 1.8 kb and long 10 kb transcripts, with the latter including a highly conserved (8.5 kb) 3′UTR, and a third additional low abundance transcript of approximately 5–7 kb [27]. MeCP2 is a nuclear protein that is mainly colocalized with densely methylated heterochromatin in mouse cells. The differential expression of *Mecp2/MECP2* transcripts can be subjected to tissue- and developmental stage-specific regulation. In the brain, differential transcript expression patterns for the two isoforms have been detected [28]. The transcript levels are high during embryogenesis with a postnatal decrease, but increasing again towards adulthood. On the other hand, the protein levels are low during embryogenesis and increase postnatally upon neuronal maturation [29].

MeCP2E1 and E2 isoforms only differ in their N-terminal sequences, sharing the functional MBD and Transcriptional Repression Domain (TRD), and it seems likely that their functional properties overlap considerably. However, several observations point towards the possibility that the two isoforms might indeed have subtle yet etiologically relevant nonredundant functions. The MeCP2E1 isoform has a putative serine residue within its N-terminus, which is absent in MeCP2E2 isoform [26]. Furthermore, differential expression of the two isoforms at the transcript level has been demonstrated in the developing mouse brain. *Mecp2E2* mRNA was enriched in the dorsal thalamus and layer V of the cerebral cortex, while more *Mecp2E1* transcripts were detected in the hypothalamus than in the thalamus between P1 and P21 [28]. Whether this reflects a similar variation of the protein expression pattern remains to be determined. Mutation analysis in RTT patients has shown that exon 1 mutations can lead to severe RTT phenotypes. Some of these mutations do not seem to affect the transcription of MeCP2E2, suggesting that MeCP2E2 alone might not be able to compensate for the loss of MeCP2E1. Although mutations in all domains of MeCP2 have been identified in RTT patients, none have been reported to be in the MeCP2E2-specific exon 2. However, a number of point mutations have been identified that are unique to the MeCP2E1, indicating that MeCP2E1-specific mutations are sufficient to cause RTT. The possibility of functional redundancy between the two isoforms has been further investigated recently by a group studying the RTT phenotype rescue capabilities of each isoform. This study showed that MeCP2E1 alone is capable of compensating for overall MeCP2 deficiency in mice, in a dose-dependent manner. While MeCP2E2 also achieved phenotypic rescue, the degree of rescue was significantly higher with MeCP2E1, even at lower dosage levels [30]. The results of this study suggest that the two isoforms have both redundant and nonredundant functions.

## 3. MeCP2 Protein Structure, Interacting Protein Partners and Posttranslational Modifications

The main functional domains of MeCP2 are the MBD, the TRD, and the C-Terminal Domain (CTD). The MBD facilitates binding to methylated CpG dinucleotides and the preference for adjacent A/T-rich motifs [31]. It is also capable of binding to nonmethylated DNA sequences such as the four-way DNA junctions [32]. However, the role of MeCP2 as a transcriptional repressor is mostly mediated through its TRD domain. The TRD interacts with corepressor complexes such as mSin3A, further recruiting HDAC1 and HDAC2, and thereby acting as a link between DNA methylation and chromatin remodelling [21]. The TRD domain further facilitates MeCP2 interaction with other partners including c-SKI [33], YY1 [34], and YB1 [35]. MeCP2 CTD is believed to have critical functions, as transgenic mice lacking MeCP2 CTD display many RTT phenotypes [36]. Recently, MeCP2 has been shown to have dual functions, also acting as a transcriptional activator *via* interaction with CREB [23], although no interacting domain has been mapped. *In vitro*, MeCP2 is known to influence chromatin compaction and nucleosome clustering [37]. In neurons, MeCP2 is also known to suppress spurious transcription of repetitive elements, thereby reducing “transcriptional noise” [38, 39].

A crucial aspect of MeCP2 that has not been fully explored is the functional effect of its Posttranslational Modifications (PTMs). Although several modifications have been detected for MeCP2, only two phosphorylation modifications have been studied in detail. Of these, phosphorylation of serine 421 (S421) is linked to neuronal activity and is known to modulate MeCP2-regulated *Bdnf *transcription [40]. Interestingly, phosphorylation of serine 80 (S80) is removed upon neuronal activity [41]. The same group detected two other phosphorylations, S399 and S424, in resting and active neurons, respectively. It would be interesting, however, to characterize any potential cross-talk between these specific PTMs in MeCP2, as seen in histones [42, 43]. Furthermore, knock-in mice models of S80 and S421/S424 showed opposing effects of the modifications on locomotor activity, implying differential function of MeCP2 based on its PTM [41]. This shows that MeCP2 PTMs enhances its capability to function dynamically within neurons, thus emphasizing the necessity of characterizing other PTMs of MeCP2.

## 4. The Expression Pattern of MeCP2

MeCP2 is widely expressed among various tissues, with higher expression in the brain. Expression studies in rodents, macaque, and humans have revealed a similar pattern of heterogeneous MeCP2 expression in brain [29, 44–49]. MeCP2 expression pattern within different brain regions follows the developmental maturation of the central nervous system, being initially detected in the earliest developing structures such as brainstem and thalamus [29, 49, 50]. In rodents, MeCP2 expression in the olfactory bulb precedes synaptogenesis [47, 48]. In general, MeCP2 expression is highest in neurons, with lower levels of the protein being detected in glia [51]. Within neurons, MeCP2 expression is lower in immature neurons and highest in postmitotic neurons [52]. The elevated levels of MeCP2 expression in mature neurons are maintained throughout adulthood, implying its importance in postmitotic neuronal function. To understand how MeCP2 deficiency impairs brain function, much effort has been focused on the neuronal cell-autonomous effects of MeCP2 deficiency, due to its high expression in mature neurons. Previous data indicate that MeCP2 deficiency in neurons is sufficient to cause RTT-like neurological phenotypes in mouse [53]. Recent studies investigating the effects of *Mecp2* deletion in specific neuronal population have observed differential phenotypic outcomes [54, 55]. These observations imply that various RTT phenotypes might be generated as a consequence of MeCP2 deficiency in specific neuronal populations. To date, a possible contribution of astrocyte dysfunction to RTT has not been fully examined, mainly due to the previous assumption that MeCP2 is not expressed in astrocytes.

In 2009, MeCP2 expression in glial cells was shown by independent groups [51, 56, 57], with significantly lower detection of MeCP2 in glia compared to neurons. MeCP2 deficient astrocytes showed functional abnormalities and were unable to support proper neuronal growth. Furthermore, MeCP2 deficient neurons were capable of exerting a nonautonomous effect on MeCP2 wild type astrocytes, and negatively regulating them. Another study on MeCP2 expression in microglia showed that MeCP2 deficiency in microglia leads to elevated secretion of glutamate and contributes to neuronal abnormalities in RTT. Perhaps the strongest evidence to support the effect of MeCP2 expression in astrocytes in RTT etiology comes from a recent study in which MeCP2 was reexpressed specifically in astrocytes of an RTT mice model. Reexpression of MeCP2 in astrocytes alone significantly improved several phenotypes including improved locomotion and prolonged lifespan. Restoration of MeCP2 on mutant astrocytes also led to a noncell autonomous effect on neighboring neurons, rescuing dendrite abnormalities and increasing the level of V-glut1 [58]. These studies show the critical role of glial cells in RTT pathology and warrant further investigation on MeCP2 function in glia.

## 5. MeCP2 Binds to Methylated DNA and Competes with Histone H1 for the Internucleosomal DNA

Eukaryotic DNA is compacted into chromatin, which is made up of nucleosome repeats [59, 60]. The nucleosome consists of a core particle composed of a histone octamer associated with DNA and a linker DNA that connects the core particles bound by one H1 linker histone. The histone octamer consists of two copies of each of the four histones H2A, H2B, H3, and H4. Histone H1 or linker histone seals two rounds of DNA at its entry/exit site on the surface of the nucleosome core and thereby stabilizes higher-order chromatin structure [61]. Histone H1 has many variants with specificity observed among species, tissue types, and even developmental stage.

Recent studies have suggested a possible relationship between histone H1 and DNA methylation [62, 63]. Microarray analysis of embryonic stem cells in which three H1 variants (H1c, H1d, and H1e) have been silenced revealed that approximately one third of the genes showing altered expression pattern were regulated by DNA methylation. A quantitative reduction in the extent of DNA methylation at specific CpG dinucleotides within the imprinting control regions of the *H19-Igf2* and *Gtl2-Dlk1* gene loci was observed in these cells. It is interesting to note that most of these genes are known to be regulated by MeCP2 (Table 1). *In vitro, *MeCP2 can compete with histone H1 and bind linker DNA [64, 65]. *In vivo*, linker H1 and MeCP2 show similar mobility in the nucleus and share the same internucleosomal binding sites, evident by Fluorescence Recovery After Photobleaching (FRAP) studies [66, 67]. Furthermore, neuronal nuclei lacking MeCP2 show 2-fold upregulation of histone H1 expression [39]. These observations suggest that in neurons, MeCP2 and histone H1 may share similar functions, at least in part with respect to chromatin organization.

## 6. MeCP2 Genomic Distributions and Target Genes

By interpreting DNA methylation, MeCP2 modulates transcriptional repression and silencing of specific target genes. In neurons, MeCP2 closely tracks the intensity of methylated DNA [39]. Recent studies on MeCP2 genomic distribution, however, indicate that it occupies both methylated and unmethylated DNA [82]. Although DNA methylation is considered to be a stable modification, it is becoming evident that in the brain, reduction of DNMTs or reduced MeCP2 association may result in decreased DNA methylation, a process previously thought to be irreversible. To fully understand the functional role of MeCP2 in the pathobiology of RTT, and to develop effective therapeutic strategies, a comprehensive knowledge of MeCP2 genomic targets is essential. To this end, several research groups have attempted to identify global gene expression alterations caused by MeCP2 dysfunction in neuronal and nonneuronal tissues from RTT patients and mice models. However, in most cases these studies have generated only a small and mostly nonoverlapping list of target genes [23, 68–80] (Table 1). Also, direct association of these identified targets towards the pathophysiology of RTT has not been established in most of these studies. One exception to this would be Brain-Derived Neurotrophic Factor (BDNF). In 2003, two independent groups demonstrated MeCP2 binding to methylated CpG sites near the promoter III of *Bdnf* in resting neurons [70, 71]. Membrane depolarization of these neurons by KCl treatment led to the dissociation of MeCP2 from the *Bdnf* promoter. Two mechanisms have been proposed for the dissociation of MeCP2: (i) reduced CpG methylation at the MeCP2 binding site following neuronal activation [70] and (ii) phosphorylation of MeCP2 at specific lysine residues [71]. Recent studies in mice models have further provided *in vivo* evidence for functional interactions between MeCP2 and *Bdnf.* Experiments on an RTT mice model have shown that knockout of *Bdnf *exacerbated the RTT phenotypes, whereas overexpression of *Bdnf *rescued a subset of RTT-like phenotypes [83]. ChIP-based experiments in neonatal mouse brain identified two MeCP2 binding sites in an imprinted gene cluster in chromosome 6 [81]. Two genes within this cluster, *Dlx5* and *Dlx6,* showed approximately twofold increases in expression, in MeCP2-null mice brain. The study also showed alterations in histone modifications and the formation of a higher-order chromatin loop at the silenced chromatin of the *Dlx5*-*Dlx6* locus in wild type and the lack of formation of the chromatin loop in RTT patients. This provided evidence for a novel mode of gene repression by MeCP2, although a similar mechanism of repression has not been shown for any other MeCP2 targets.

## 7. Human Diseases Associated with *MECP2* Mutations

MeCP2 mutations are mostly sporadic, occurring preferentially as C > T transitions of CpG dinucleotides and mostly on the paternal X chromosome [84, 85]. As mentioned earlier, *MECP2* mutations are mainly associated with Rett Syndrome, a progressive postnatal neurological disorder predominantly affecting females with an incidence of 1 in 10,000 [86]. RTT is characterized by an apparently nonsymptomatic phase for the first 6–18 months of age followed by apraxia, deceleration of head growth, gait abnormalities, stereotypic hand movements, and mental retardation. The lifespan of RTT patients is variable, and some patients survive up to 70 years of age [87, 88]. In male individuals, *MECP2* mutation leads to fatal neonatal encephalopathy [89], Rett syndrome-like features, and familial X-linked mental retardation with or without motor abnormalities [89–91]. Male patients with RTT usually have a short lifespan and very often develop congenital encephalopathy [92, 93]. *MECP2* mutations have been detected in more than 90% of classical RTT patients. Approximately 65% of *MECP2* mutations causing RTT can be attributed to 8 recurrent missense or nonsense mutations within the MBD (R106W, R133C, T158M, and R168X) or TRD (R255X, R270X, R294X, and R306C) [94, 95].

Previous studies have implicated possible correlations between these mutations and RTT phenotypes [14, 96]. MeCP2 mutations have also been detected, albeit in very few patients, with Prader-Willi syndrome [97], Angelman syndrome [98], nonsyndromic mental retardation [99], and autistic patients [100]. Currently, Rett Syndrome has no effective treatment. However, in RTT mice lacking *Mecp2*, reactivation of the *Mecp2* gene after the onset of disease can rescue the disease phenotype [101, 102]. This demonstrates the possibility of RTT gene therapy strategies, where delivering *MECP2 *into the affected neurons may indeed improve RTT symptoms. Creating the first generation of *MECP2* isoform-specific retroviral and lentiviral gene therapy vectors, we showed their efficient and long-term expression in the adult brain-derived neural stem cells, in their neuronal progenies, and in the brain microenvironment [56]. However, the *in vivo* rescue effect of the gene therapy delivery of these viruses remains to be elucidated. Our studies also showed the feasibility of using the endogenous *Mecp2 *promoter for transgenic *MECP2* expression. This is significant, since one of the critical concerns towards the design of RTT gene therapy strategy is the prevention of *MECP2* overexpression. In humans, overexpression of *MECP2* caused by duplication of the *MECP2 *locus leads to a variety of neurological symptoms including seizures and mental retardation [103–105]. Alternatively, drug treatments can be designed to target proteins, which may compensate for MeCP2 loss in neurons. One study, in particular, has provided great hope towards pharmacological treatment of RTT in the future. Treatment of an RTT mice model with the active peptide fragment of IGF-1 significantly improved many disease phenotypes and extended the overall lifespan of the mice [106]. The generation of RTT-specific iPS (induced Pluripotent Stem) cells has provided an ideal platform to analyze potential pharmacological treatments for Rett Syndrome [107, 108].

## 8. Closing Remarks

One of the most studied genes to link epigenetics to human disease is the X-linked *MECP2* gene. *MECP2 *mutations lead to Rett Syndrome and are also associated with a broad spectrum of neurological disorders. Despite the impressive progress on our understanding about MeCP2, there are still many fundamental questions remaining to be addressed; at the methylated DNA, does MeCP2 dimerization require hetero- or homodimerization? Do MeCP2 isoforms show differential expression and/or function in CNS and are they developmentally regulated? What are the factors that regulate MeCP2 expression and splicing within various tissues? And finally, does MeCP2 act as a nonspecific DNA methyl binding protein on methylated DNA or does it recognize and prefer particular sites within the genome and what is the contribution of its interacting protein partners towards defining the specificity and sensitivity of such genomic distribution? A comprehensive knowledge of these unanswered questions will help to understand how the products of a single gene, such as *MECP2*, have such vast functional properties.

## Figures and Tables

**Table 1:** Known targets of MeCP2.

Gene target	Function	Cell/tissue type studied	Direct association with MeCP2 (cell line used for ChIP)	Reference
*PCDHB1*	Cell adhesion	Oral cancer cell lines (ZA, KOSC2, HSC5, NA)	Yes (SH-SY5Y)	
*PCDH7*	Cell adhesion		Yes (SH-SY5Y)	[68]
*APBP3*	Intracellular signal transduction		Yes (SH-SY5Y)	

*CLU*	Extracellular molecular chaperone		No (SH-SY5Y)	
*CRMP1*	Component of semaphoring signal transduction pathway		Yes (SH-SY5Y)	
*DNMI*	Vesicular trafficking, production of microtubule bundles, hydrolyzes GTP		Yes (SH-SY5Y)	[69]
*GNBI*	Integrates signals between receptor and effector proteins	RTT patient brain (frontal cortex)	Yes (SH-SY5Y)	
*APLP1*	Enhancer of neuronal apoptosis		No (SH-SY5Y)	
*CO1*	Mitochondrial respiratory chain		No (SH-SY5Y)	
*GDI1*	Regulates GDP/GTP exchange		No (SH-SY5Y)	

*Bdnf*	Neuronal plasticity and survival	Mouse E14 cortical culture Rat E18 cortical neurons	Yes (mouse E14 cortical culture Rat E18 cortical Neurons)	[70, 71]

*Fxyd1*	Ion transport regulator for Na, K-ATPase	RTT mice cerebellum RTT patient's brain—superior frontal gyrus	Yes (adult mice brain, *Mecp2* wt and *Mecp2* null mouse; HEK293T cells)	[72, 73]

*Reln* *Gtl2*	Neuronal layer formation, cell-cell interactions Growth suppressor	RTT mice cerebellum	Yes (adult mice brain)	[72]

*ID1* *ID2* *ID3* *ID4*	Regulation of neuronal differentiation	SH-SY5Y	Yes (SH-SY5Y)	[74]

*IGFBP3*	Modulation of IGF functions	RTT mice model	Yes (HeLa cells; mice cortices)	[75]

*UBE3A* *GABRB3*	Ubiquitin ligase GABA-A receptor	Brain cerebral samples of RTT, AS, and autism patients	No (adult mouse cerebellum samples)	[76]

*Sst* *Oprk1* *Gamt* *Gprin1* *Mef2c* *A2bp1*	Regulation of cell migration Signal transduction Organ morphogenesis Neurite development Neuron development and differentiation RNA splicing and mRNA processing	RTT mice models (*Mecp2* null and *Mecp2* Tg) and control mice; Hypothalamus	Yes (RTT mice models (*Mecp2* null and *Mecp2* Tg) and control mice; Hypothalamus)	[23]

*xHairy2a*	Neuronal differentiation	Xenopus embryos	Yes (xenopus neurula stage embryos)	[77]

*Sgk1* *Fkbp5*	Cellular stress response Hormone signalling	RTT mice model; brain samples	Yes (mice brain tissue)	[78]

*Uqcrc1*	Mitochondrial respiratory chain	RTT mice model; brain samples	Yes (adult mice; whole brain)	[79]

*Crh*	Stress response	RTT mice model; brain samples	Yes (RTT mice brain samples)	[80]

*Dlx5*	Transcription factor	Not done	Yes (mouse brain)	[81]
